# Advance in chimeric antigen receptor T therapy in autoimmune diseases

**DOI:** 10.3389/fimmu.2025.1533254

**Published:** 2025-03-04

**Authors:** Xiaolan Ji, Yunfan Sun, Yuyang Xie, Jianling Gao, Ji Zhang

**Affiliations:** ^1^ Department of Ophthalmology, The Second Affiliated Hospital of Suzhou University, Suzhou, China; ^2^ The First Clinical College of Nanjing Medical University, Nanjing, China; ^3^ The First Clinical Medicine School, Suzhou Medical College, Soochow University, Suzhou, China; ^4^ Department of Critical Care Medicine, The Fourth Affiliated Hospital of Soochow University, Suzhou, China

**Keywords:** CAR-T, autoimmune diseases, immunotherapy, CAR-Treg, CAAR-T

## Abstract

Autoimmune diseases are a group of diseases in which the body’s immune system misrecognizes its own antigens resulting in an abnormal immune response, which can lead to pathological damage to or abnormal functioning of its own tissues. Current treatments are mainly hormones and broad-spectrum immunosuppressants, but these can lead to a decline in the patient’s immunity. Chimeric antigen receptor T (CAR-T) Cell therapy has emerged, and now the structure of CAR has changed from first generation to fourth generation of CAR. The significant achievement of CAR-T therapy to B-cell leukemia has also inspired the treatment of autoimmune diseases, and by investigating the mechanisms of different autoimmune diseases, different designs of CAR-T can be used to specifically treat autoimmune diseases. In this review, we will discuss the therapeutic strategies of CAR-T cells in different autoimmune diseases and the limitations of the treatment.

## 1 Introduction

CAR⁃T cells are a group of T-cells that express a multifunctional synthetic receptor through gene editing technology. CAR-T cell therapy is a method of obtaining CAR-expressing T cells by introducing exogenous artificially-designed CAR genes into T cells for genetic modification and transformation (1), and then infusing these cells back into patients for treatment after *in vitro* scale-up and expansion. CAR-T cells are capable of specifically recognizing and killing tumor cells through the expression of CAR, which belongs to the category of precision tumor immunotherapy. Since Gross first introduced the concept of CAR in 1989 (2), the CAR structure has been gradually improved through continuous research.

**Figure 1:**
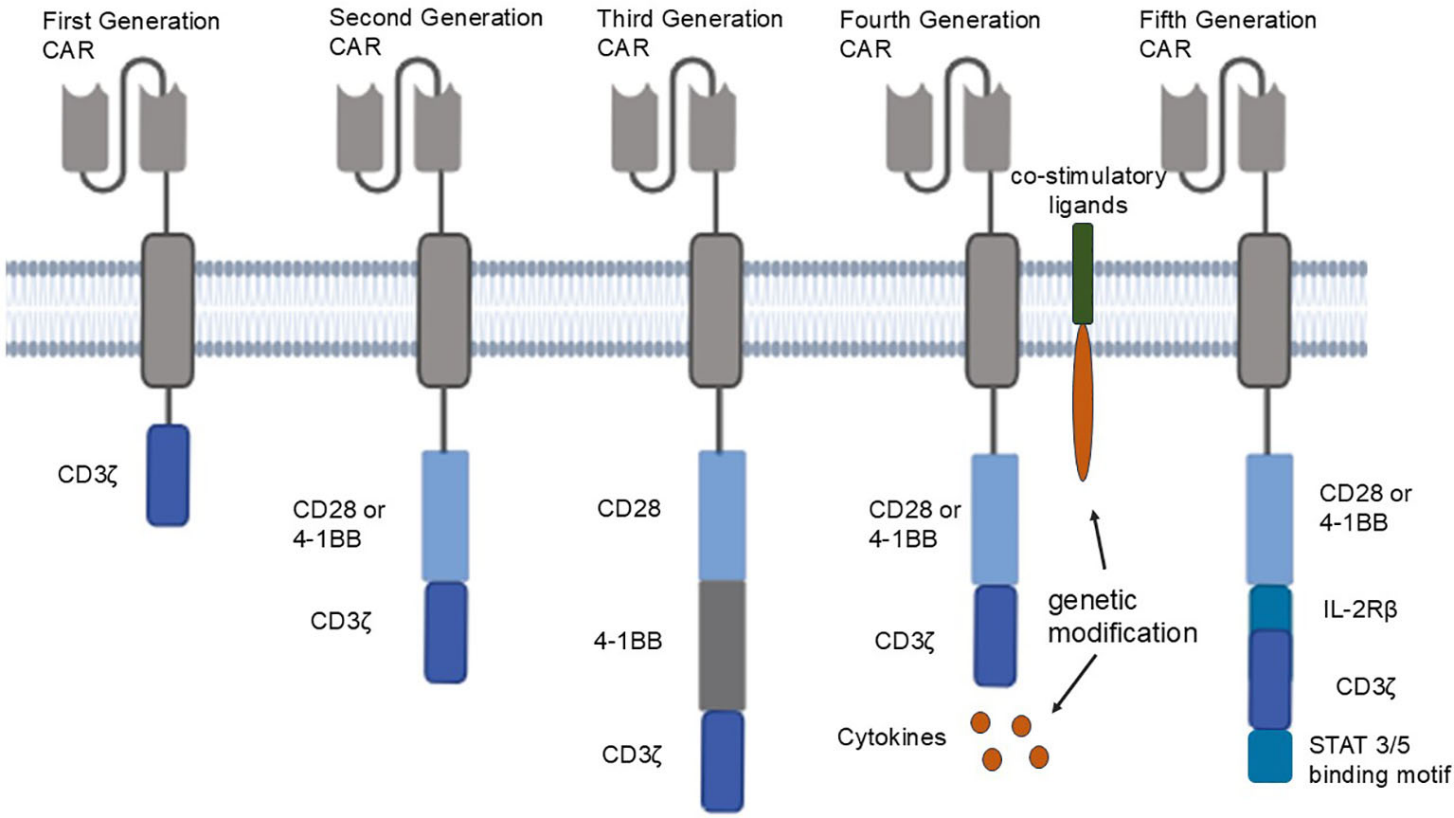
From left to right are the five generations of CAR structures: first generation CAR: first generation CAR expresses a single-chain fragment variable (scFv) segment recognizing a specific molecule extracellularly, and a CD3ζ signaling domain in tandem intracellularly; second generation CAR adds a CD28 or 4-1BB co-stimulatory signaling domain to first generation CAR; third generation CAR combines CD28 and 4-1BB, or CD28 and OX-40, to second generation CAR; fourth generation CAR secretes cytokines or expresses co-stimulatory ligands through genetic modification; fifth generation CAR adds co-stimulatory domains that activate other cytokine signaling between CD28 and CD3ζ.

Nowadays, the intracellular region of CAR T cells has evolved from the first-generation CAR containing only one CD3 signaling molecule to the fifth generation CAR structure containing multiple signaling molecules (**Figure 1**). First generation of CAR structures expresses single-chain fragment variable (scFv) segments recognizing specific molecules extracellularly, and intracellularly tandem with a CD3ζ signaling domain. The addition of a CD28 or 4-1BB co-stimulatory signaling domain to the intracellular structure of the second generation CAR structure has led to significant success of second generation CAR⁃T cells in the treatment of refractory or relapsed B-cell leukemia or lymphoma (3). The third generation CAR structure combines CD28 and 4-1BB or CD28 and OX-40 to enhance T-cell efficacy based on the second generation CAR structure (with two co-stimulatory domains) (4). The fourth generation of CAR T cells is known as T cells redirected for universal cytokine killing (TRUCKs) (5), expressing of cytokines or co-stimulatory molecular ligands to eliminate antigen-negative tumor cells from tumor tissues by releasing inducible cytokines that promote T-cell activation and recruit and activate intrinsic immune cells. The fourth-generation CAR⁃T cells also play well in solid tumors that were difficult to address in the first three generations (6). In recent years, fifth generation CAR has been proposed, which adds co-stimulatory structural domains that activate other cytokine signaling, such as intracellular IL-2 receptor β-chain (IL-2Rβ) structural domains and STAT3/5 structural domains, between CD28 and CD3ζ on the basis of the structure of the second generation CAR. This enables fifth generation CAR to provide antigen-dependent cytokine signaling, enhance the proliferation and survival of CAR-T cells, and improve the anti-tumor activity of CAR-T cells (7, 8).

CAR-T cell technology has now achieved great success in the treatment of hematological tumors, and preliminary clinical studies (9–11) have shown that CAR-T (CD19-CAR-T) cells specifically targeting the CD19 molecule can be effectively targeted for the treatment of B-cell malignancies, including relapsed and refractory B acute lymphoblastic leukemia and B-cell lymphoma. Compared with the current conventional treatments for hematological malignant diseases, CAR-T cell therapy is more effective than chemotherapy. Compared with hematopoietic stem cell transplantation, CAR-T cells are inexpensive and do not require mating, which is a very obvious advantage. In recent studies, CAR-T also has a regulatory effect on plasma cells and can target specific markers of plasma cells to remove antibodies produced by plasma cells, thus improving the survival of car-t cells and the clinical efficacy of CAR-T cell therapy. But now further experimental verification is still needed. But in general, CAR-T cell therapy has already become a powerful weapon in the fight against hematological tumors.

Autoimmune disease (AID) refers to a group of diseases in which the human immune system misrecognizes its own antigens, resulting in an abnormal immune response that can lead to pathological damage to its own tissues or abnormal function. According to the scope of autoimmune response, it is mainly divided into organ-specific AID and systemic AID (12).

## 2 Chimeric antigen receptor T cells therapeutic strategy for autoimmune diseases

The pathogenesis of AID is complex, and clinical manifestations and diagnostic methods vary. Currently, the main treatments are hormones and broad-spectrum immunosuppressants, both of which work by suppressing the immune system, but the patients’ own immune function decrease after treatment, and it is not possible to retain the normal function of the immune system for a long period of time or even permanently. B cells play an important role in the development of some autoimmune diseases, including antigen presentation, activation of T cells, and production of autoantibodies (13). Therefore, maintaining the stability of immune cells in the body has a significant place in the treatment of autoimmune diseases, and the success of CAR T-cell therapy in the treatment of hematologic tumors such as B-lymphoblastic leukemia (14) has also given significant inspiration to treat autoimmune diseases.

**Figure 2:**
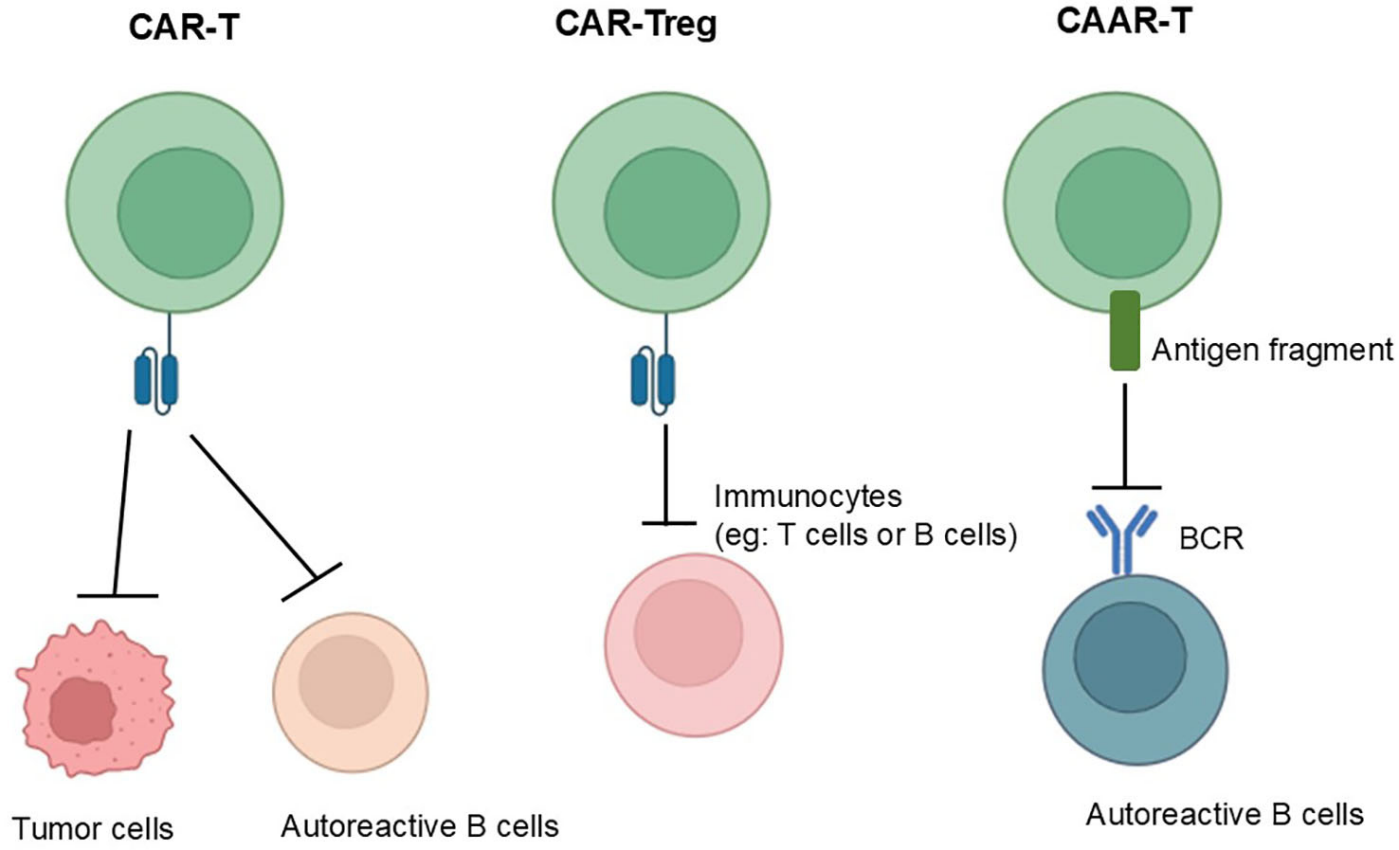
From left to right, the mechanisms of action of conventional CAR, CAR-Treg and CAAR-T are shown, with the mechanism of conventional CAR-T shown on the far left: which can accurately identify and attack targeted cells; in the middle is the mechanism of action of CAR-Treg: which can suppress effector T-cells *in vitro* and *in vivo* by introducing chimeric antigen receptor T-cells that inhibit over-activated immune responses by modulating immune cells; and the far right shows the mechanism of action of CAAR-T: which can target immune cells that produce specific autoantibodies, thereby reducing autoantibody titer and inhibiting over-activated immune responses.

There are now three main CAR-T treatment strategies: classical CAR-T; CAR-Treg (**Figure 2**); Chimeric Autoantibody Receptor-T (CAAR-T) Cells. Classical CAR-T cell therapy enables T cells introduced with chimeric antigen receptors to accurately identify and attack targeted cells, such as abnormal autoimmune factors, resulting in a reduction of over-activated autoimmune cells and enabling precision therapy by deeply analyzing the pathogenesis of disease; CAR-Treg cell therapy is capable of localizing to areas of immune abnormality and suppressing effector T-cells *in vitro* and *in vivo* by introducing chimeric antigen receptor T-cells that inhibit over-activated immune responses by modulating immune cells; CAAR-T cells can target immune cells that produce specific autoantibodies, thereby reducing autoantibody titer and inhibiting over-activated immune responses.

The table below summarizes three CAR-T-based therapies.

### 2.1 Classical CAR-T immunotherapy

#### 2.1.1 Therapy in systemic lupus erythematosus (SLE)

B cells play an important role in the development of SLE, including the presentation of antigens, activation of T cells, and production of autoantibodies (13), reducing the persistence of autoantibody-secreting plasma cells and memory B cells in SLE patients is therefore a key to treating SLE. In 2021, Jin et al (17) demonstrated in a mouse model of SLE that administration of CD19-targeted CAR-T therapy resulted in depletion of B-cells, interruption of autoantibody production, and reversal of glomerulonephritis and other organ damage in mice. In September 2022, Prof. Georg Schett’s team at the University of Erlangen-Nuremberg, Germany, reported in Nature Medicine the results of CAR-T therapy in five patients with refractory SLE (15). The five patients who were treated with CAR-T had no anti-dsDNA antibodies and an overall reduction in autoantibodies; they did not relapse during the 17-month follow-up period, and all of them achieved drug-free DORIS (the definitions of remission in SLE) remission without receiving any medication related to SLE during the treatment period (16). The results showed that all SLE patients achieved DORIS remission at 6 months post-treatment, with significant clinical responses in patients with idiopathic inflammatory myopathies and reduced disease activity in patients with systemic sclerosis. Grade 1 cytokine release syndrome (CRS) occurred in 10 patients, and 1 patient was hospitalized with grade 2 CRS, grade 1 immune effector cell-associated neurotoxicity syndrome, and pneumonia (**Table 1**).

**Table 1:** This table summarizing various CAR-T-based therapies, including CAR-T, CAR-Treg and CAAR-T, which details the target antigen, mechanism of action, associated diseases, and links to relevant clinical or preclinical studies.

	Target	Mechanism of action	Associated diseases	References
**CAR-T**	CD19	deplete B cells, block autoantibody production	systemic lupus erythematosus (SLE)	(15–17)
	BCMA	broad immunosuppression	myasthenia gravis (MG)	(18)
	mAb287	maintains specificity of mAb287 and kill antigens	type 1 diabetes(T1D)	(19)
**CAR-Treg**	CEA and TNP	Maintain Foxp3 level, repeat expansion and specifically suppress Teff cells	ulcerative colitis (UC)	(20–22)
	mIgE	recognize and bind to lgE-expressing cells, produce cytotoxicity	allergic asthma (AA)	(23, 24)
	Th17 and CV	Reduce the number of inflammatory Th17 cells and specific identify CV or CV+ cells	rheumatoid arthritis (RA)	(25–27)
**CAAR-T**	Dsg3	exhibit specific toxicity to Dsg3 BCR-expressing B cells, decrease the titer of Dsg3 autoantibodies	pemphigus vulgaris (PV)	(28)
	MuSK	Induces B-cell cytotoxicity against B-cells expressing anti-MuSK surface self-antigens	muscle-specific tyrosine kinase myasthenia gravis (MuSK MG)	(29)
	ds-DNA	Improve organoid morphology, apoptosis and inflammatory processes in IFNα-stimulated anti-dsDNA+ B cells	lupus nephritis (LN)	(30)

Classical CAR-T cell therapy enables T cells introduced with chimeric antigen receptors to accurately identify and attack targeted cells, such as abnomal autoimmune factors, resulting in a reduction of over-activated autoimmune cells and enabling precision therapy by deeply analyzing the pathogenesis of disease; CAR-Treg cell therapy is capable of localizing to areas of immune abnormality and suppressing effector T-cells *in vitro* and *in vivo* by introducing chimeric antigen receptor T-cells that inhibit over-activated immune responses by modulating immune cells; CAAR-T cells can target immune cells that produce specific autoantibodies, thereby reducing autoantibody titer and inhibiting over-activated immune responses.

#### 2.1.2 Therapy in myasthenia gravis (MG)

MG is an autoimmune disease characterized by autoantibody-mediated impairment of acquired neuromuscular junction (NMJ) transmission, mainly caused by anti-acetylcholine receptor (AChR) antibodies (31). B cells play a crucial role in the pathologic process of MG (32). Descartes-08 is a CAR ⁃T therapy that expresses CAR from mRNA, transiently rather than permanently, thereby reducing the risks inherent in traditional CAR ⁃T cell therapy, and targets BCMA for the treatment of generalized myasthenia gravis (gMG). Recently, Tahseen Mozaffar et al. published the results of a phase Ib/IIa clinical trial of Descartes ⁃08 in patients with gMG (18), in which the first cohort of three patients with gMG showed significant disease improvement and tolerated the drug well, with no CRSs or other serious adverse effects. The second cohort 11 gMG patients had no dose-limiting toxicity, CRS, or neurotoxicity at a median follow-up of 5 months (range, 3 to 9 months) and showed significant improvement in MG clinical performance at up to 9 months post-infusion.

#### 2.1.3 Therapy in type 1 diabetes(T1D)

Type 1 diabetes (T1D) is an autoimmune disease in which pancreatic B-cells are damaged by a variety of factors (autoimmunity, genetics, viral infections, etc.) and fail to secrete insulin normally. A variety of abnormal autoantibodies can be produced in patients, such as anti-glutamic acid decarboxylase antibodies, insulin antibodies, islet cell antibodies, etc. The most common treatment for T1D is the injection of artificial insulin preparations, but this does not provide a cure. Zhang et al. (19) used mAb287 chimeric antigen receptor T (287⁃CAR⁃T) cells and found that they could temporarily inhibit autoimmunity against pancreatic islet cells and delay the onset of T1D in untreated mice by about 6 weeks. They found that insulin autoantibodies were detectable in both control mice at week 10, but not in the 287⁃CAR⁃T cell-treated mice. However, they found that the efficacy of 287-CAR-T cell therapy was not long-lasting, and the 287-CAR-T cells in the mice gradually decreased over time, becoming undetectable by 25 weeks. This suggests that the current single-injection CAR-T cell therapy can only delay the progression of diabetes, but not stop it.

### 2.2 CAR-Treg immunotherapy

#### 2.2.1 Therapy in ulcerative colitis (UC)

Ulcerative colitis is an inflammatory bowel disease characterized by mucosal inflammation that begins at the distal end, gradually expands proximally, and eventually involves the entire colon, often with recurrent episodes. The key cause of its pathogenesis is the imbalance in the dynamic balance between the intestinal flora and the body’s immune response. Reduced activity of peripheral regulatory T cells (Treg) leads to over-activation of intestinal immune responses. Elinav et al (20) proposed that natural Tregs (nTreg) can efficiently express functional, antigen-specific chimeric receptors, and they found that nTreg with a variable region of antibodies against 2, 4, 6 ⁃ Trinitrophenol (TNP) specifically inhibited effector T cells *in vitro* and *in vivo (* 21), and in mice model of colitis, the cells significantly ameliorated the symptoms of colitis in mice. Blat et al (22) evaluated the ability of CAR⁃Treg to redirect to the inflammatory part of the colon and the regulation of CAR⁃Treg. They used carcinoembryonic antigen (CEA), which is overexpressed in colitis, as the target of CAR⁃Treg targeting, and designed a chimeric embryonic antigen receptor-regulated T cell (CEA-CAR⁃Treg) to target the inflammatory part of the colon. The results show that CEA⁃CAR⁃Treg can be redirected to the site of inflammation to suppress the immune response. This study also found that CAR⁃Treg has more potent inhibitory activity than polyclonal Treg. More importantly, CEA⁃CAR⁃Treg can inhibit the progression of colitis to colorectal cancer, and its use in the early stages of colitis significantly reduces the risk of colorectal cancer progression. These studies have shown that CAR⁃Treg is effective in animal experiments (33). It is important to note that the effects of CAR⁃Treg are also influenced by the microenvironment, and some microenvironments may even mediate the opposite effect of CAR⁃Treg. If Treg loses expression of the forehead box P3 (FOXP3) in an inflammatory environment, the immunosuppressive phenotype of Treg is altered. Treg can even exhibit effector cell properties (34), secreting proinflammatory cytokines to exacerbate inflammation. Therefore, it is necessary to design a specific CAR⁃Treg for the specific microenvironment to ensure safety and effectiveness (35).

#### 2.2.2 Therapy in allergic asthma (AA)

Asthma is a common chronic inflammatory disease of the respiratory tract, and eosinophils, as the main inflammatory effector cells in asthma, and lgE, a key mediator mediating the allergic response, are one of the main targets for the treatment of asthma. Skuljec et al. (23) studied an experimentally induced asthma mouse model and found that CAR⁃Treg redirected to the lungs and aggregated in the lungs and tracheobronchial lymph nodes of the mice, significantly attenuating airway inflammation and infiltration of inflammatory cells in the lungs compared to the control group. Ward et al. (24) used the highly specific binding between Fc epsilon receptor I (FcϵRI) and IgE to design a CAR with FcϵRIα as the extracellular region of the receptor, which specifically recognizes and binds to lgE-expressing cells and produces cytotoxic effects. However, high concentrations of secreted lgE were found to inhibit the binding of these CAR⁃ T cells to target cells. To address this issue, they used CAR⁃T cells with a low affinity mutant of FcϵRIα, and found that they could tolerate high concentrations of lgE and at the same time produce cytotoxicity against mIgE-expressing myeloma cells *in vitro*. Low affinity mutant CAR has very high affinity for mast cells expressing membrane bound isoform of IgE (mIgE) (36).

#### 2.2.3 Therapy in rheumatoid arthritis (RA)

Rheumatoid arthritis (RA) is an autoimmune disease of unknown etiology, and one of the major causes of its development is a decrease in the ability of Treg to block the production of interferon γ (IFN ⁃ γ) and tumor necrosis factor α (TNF ⁃ α) by CD4 + CD25 + T cells (37). Developing specific CAR⁃Tregs to induce immune tolerance in the affected synovium is also a promising treatment strategy for RA. Wright et al. (25) utilized ovalbumin (OVA)-specific Tregs to inhibit OVA-induced arthritis, including T cell receptor (TCR)-transduced primary Tregs and TCR⁃FoxP3-transduced CD4+ T cell-induced Tregs, both modified Tregs reduce the proliferative function of different antigen-specific T cells through bystander inhibition. TCR⁃Tregs and TCR⁃FoxP3-induced Tregs localized to damaged tissues, reduced the number of inflammatory Th17 cells, and significantly reduced arthritic bone destruction. Citrullinated vimentin (CV) is a modified protein found only in rheumatoid arthritis, and CV⁃CAR⁃Treg can specifically (26) recognize CV or CV+ cells in patients with RA, thus improving the specific localization of Treg. CV-CAR⁃Treg enhances the specific blocking function of Treg (27), making it a promising new treatment for RA in the future.

### 2.3 CAAR-T immunotherapy

#### 2.3.1 Therapy in pemphigus vulgaris (PV)

Pemphigus vulgaris (PV) is an autoimmune herpetic disease whose pathogenesis involves autoantibodies directed against pemphigus 1 and pemphigus 3 (Dsg3). These antibodies disrupt bridging granules and interfere with the cohesion between keratinocytes in the epidermis, causing damage to healthy skin function and the formation of blisters and ulcers on the skin or mucous membranes. To address this pathogenesis, Ellebrecht et al. (28) designed a CAAR⁃T cell therapy that targets B cells producing Dsg3-specific autoantibodies. In cultured cells *in vitro*, Dsg3-CAAR-T cells showed specific toxicity to B cells expressing the Dsg3 BCR, reducing the titer of the Dsg3 autoantibody. In addition, studies in PV mouse models have shown that Dsg3-CAAR-T cells can persist *in vivo* and target Dsg3-specific B cells. This Dsg3⁃CAAR⁃T cell therapy is designed to selectively target and kill Dsg3 antibody-producing B-cells while preserving healthy B-cells critical for immune function. However, data from the phase I clinical trial of Dsg3 ⁃CAAR ⁃T cells in patients with mucosal PV (NCT04422912) have not yet been published, and further research and clinical trials are needed that will help to evaluate the potential of CAAR ⁃T in the treatment of PV.

#### 2.3.2 Therapy in muscle-specific tyrosine kinase myasthenia gravis (MuSK MG)

Like MG, muscle-specific tyrosine kinase myasthenia gravis (MuSK MG) is an autoimmune disease that causes life-threatening muscle weakness (38, 39) due to anti-MuSK autoantibodies that disrupt neuromuscular junction signaling. There are some differences in the treatment of myasthenia gravis between MuSK antibody-positive and acetylcholine receptor (AchR) antibody-positive myasthenia gravis. To avoid chronic immunosuppression caused by current therapies, Sangwook et al. (29) designed T cells expressing chimeric autoantibody receptor MuSK with CD137-CD3ζ signaling structural domains (MuSK-CAART) to precisely target B cells, which expressing anti autoantibodies. In an experimental autoimmune MG mouse model, MuSK-CAART reduced the IgG levels of anti-MuSK, but did not reduce B-cell levels or total IgG levels. No specific off-target interactions of MuSK-CAART were identified *in vivo*, in primary human cell screens, or by high-throughput human membrane proteome arrays. These data contribute to the design of new drug applications and phase 1 clinical trials for MuSK-CAART as a treatment for MuSK autoantibody-positive MG.

#### 2.3.3 Therapy in lupus nephritis (LN)

Lupus nephritis is one of the complications of SLE. LN is present in 40% to 60% (40, 41) of SLE patients at initial presentation, with a recurrence rate of 33% to 40% and progression to end-stage renal disease in about 20% of patients (42). Several autoantibodies, particularly against double-stranded DNA (anti-dsDNA), are believed to play an important role in inducing glomerular inflammation (30, 43, 44). Currently, glucocorticoids and immunosuppressive agents are the mainstay of LN treatment, and although traditional treatment programs based on these agents can improve the symptoms and prognosis of LN, adverse drug reactions and disease recurrence have prompted the search for new therapies (40).

To address the challenge of chronic immunosuppression associated with current therapies, Cristina et al. (45) designed T cells expressing a chimeric autoantibody receptor (DNA-CAART) that precisely targets B cells, which expressing anti-dsDNA autoantibodies. Depending on the antigenic specificity (including α-actinin, histone-1, heparan sulfate, or C1q), they transduced T cells from LN patients using six different CAAR vectors. The cytotoxicity, cytokine production and cell-to-cell contact of DNA-CAART were demonstrated in co-culture experiments with B cells (anti-dsDNA-positive and non-dsDNA-positive) isolated from patients. DNA-CAART was intensively investigated in co-culture experiments with B cells (anti-DNA-positive and non-DNA-positive) isolated from patients. Its efficacy was further evaluated *in vitro* immunologic renal LN organ model. Among the six proposed DNA-CAART, DNA4 and DNA6 exhibited excellent selective cytotoxic activity against anti-dsDNA^+^ B cells. In particular, DNA4-CAART ameliorated organoid morphology, apoptosis and inflammatory processes in the presence of IFNα-stimulated anti-dsDNA^+^ B cells. These findings suggest that DNA4-CAART is a promising candidate for modulating autoimmunity and may be a novel approach for the treatment of LN.

## 3 Improvements

In the last decade, CAR⁃T cell therapy has achieved remarkable efficacy in hematological oncology, and at the same time, more researchers have attempted to use CAR T to treat autoimmune diseases, which has shown very good potential in numerous experiments, but still faces greater challenges and improvements. First, CAR-T cell therapy still faces challenges such as high cost, time-consuming production process and the inherent risk of manufacturing failure, immune-related adverse events, and the risk of relapse/refractoriness (46). Secondly, how to improve T cells enrichment efficiency, maintain the long-term survival of CAR⁃T cells *in vivo*, increase the memory phenotype of CAR⁃T cells, and reduce the depletion phenotype of CAR⁃T cells also need to be explored in subsequent experiments. Thirdly, when developing CAR cell-based therapies for autoimmune diseases, it is also important to take into account the special features like off-target effects, the complexity of clinical implementation, and the risks of immunosuppression. Last, existing studies have demonstrated that CAAR ⁃T cells and CAR ⁃Treg cells can play a significant role in AID model. However, nowadays CAR-Treg and CAAR-T need further research. The application of CAAR-T cells is limited to antibody-mediated AID produced by autoimmune B cells. The development of CAR⁃Treg is faster than that of CAAR⁃T cells, because CAR⁃Treg can better solve the fundamental problem of AID, i.e., the loss of self-tolerance in autoimmune diseases, and it has become a hot spot of research.

## 4 Conclusions

Unlike the characteristic molecules that are relatively easy to target in hematologic tumors (e.g., CD19, CD20, BCMA, etc.), in the vast majority of autoimmune diseases, the specific autoantibody-mediated antigenic targets are still unknown. In some cases, exposure to autoantigenic epitopes or cross-reactivity, for example, produces autoantibodies that are inappropriately targeted to such antibody-bound antigenic epitopes, and there is overlap in the autoantibody profiles of many autoimmune diseases. This makes it more difficult to identify those autoantigenic structures that can be targeted by CARs (47). Unlike normal T cells in tumor patients, T cells in patients with autoimmune diseases often have abnormal function. The use of these abnormal T cells in the preparation of CAR⁃T may also have an impact on the efficacy of CAR⁃T, which can be improved by gene editing technology, is crucial. Currently, the application of CAAR⁃T cells is limited to antibody-mediated AID produced by autoimmune B cells, and its scope of application is relatively narrow. The development of CAR-Treg is faster than that of CAAR⁃T cells because CAR-Treg can better solve the fundamental problem of AID, i.e., the loss of self-tolerance, and now CAR-Treg has become a hot spot of research. Future research directions should focus on finding specific antigens to construct antigen-specific CARs, making CARs more specific. However, finding the self-antigenic target is not an easy task, and a large number of experiments are needed to keep exploring it. In addition, the key to the question of the durability of CAR-T cells is to find co-stimulatory domains with stronger vivo and vitro functionality and durability, and more attention will need to be paid to the combination of different co-stimulatory domains in future studies. Although a large number of studies have made considerable progress, there is still a long way to go to achieve durable, specific and safe clinical treatment.
